# Residential Environmental Composition and Risk of Acute Cellular Rejection After Heart Transplantation: A Multi-Scale Buffer Analysis

**DOI:** 10.3390/jcm15093272

**Published:** 2026-04-24

**Authors:** Tomasz Urbanowicz, Krzysztof Skotak, Dominika Konecka-Mrówka, Rafał Skowronek, Jakub Bratkowski, Jerzy Nożyński, Julia Gierszewska, Jarosław Bartkowski, Hanna Wachowiak-Baszyńska, Piotr Przybyłowski, Marek Jemielity

**Affiliations:** 1 Cardiac Surgery and Transplantology Department, Poznan University of Medical Sciences, 61-107 Poznan, Poland; 2 Institute of Environmental Protection—National Research Institute, 02-170 Warsaw, Poland; 3 Department of Histopathology, Silesian Centre for Heart Disease, Medical University of Silesia in Katowice, 41-800 Zabrze, Poland; 4 Student Research Group, Medical Faculty, Poznan University of Medical Sciences, 61-107 Poznan, Poland

**Keywords:** heart transplantation, acute cellular rejection, environment, land-use, buffers, geospatial analysis

## Abstract

**Background:** Acute cellular rejection (ACR) after heart transplantation remains incompletely explained despite standardized immunosuppression. Environmental exposures may contribute to residual immune activation; however, prior studies have focused primarily on air pollution rather than residential land-use composition. **Objectives:** To determine whether buffer-specific residential environmental composition is associated with rejection risk and whether these associations are scale-dependent and domain-specific. **Methods:** In this retrospective single-center cohort study, 30 heart transplant recipients contributed 267 biopsy-linked observations. Residential land-use composition was quantified within 300 m, 500 m, 700 m, and 1000 m buffers and aggregated into five domains: trees, other green surroundings, roads, water, and industrial land. Associations with ACR were evaluated using clustered logistic regression models adjusted for time since transplantation. **Results:** The strongest and only statistically robust associations after FDR correction were observed within the 300 m buffer. Tree-dominant (OR 1.42, 95% CI 1.22–1.65, q = 0.010) and industrial land exposure (OR 1.50, 95% CI 1.28–1.76, q = 0.010) were independently associated with increased odds of ACR. At 500 m, the association with trees persisted nominally (OR 1.39, 95% CI 1.03–1.88, *p* = 0.034), but did not remain significant after FDR correction, whereas water exposure showed a non-significant trend (OR 1.28, *p* = 0.057), which did not reach statistical significance. No associations were observed beyond 700 m across all models. **Conclusions:** Residential environmental composition may be associated with acute cellular rejection after heart transplantation in a scale-dependent manner, with signals confined to the immediate residential environment. Tree-dominant exposure within 300 m showed an association in clustered models; however, this finding was attenuated in mixed-effects sensitivity analyses. These results should be considered exploratory and hypothesis-generating study.

## 1. Introduction

Despite major advances in contemporary heart transplantation, acute cellular rejection (ACR) remains a defining event in the early post-transplant course and continues to shape longer-term graft health [1,2,3]. Even when clinically overt episodes are controlled, cumulative rejection burden contributes to subsequent allograft injury and later adverse remodeling, including cardiac allograft vasculopathy. What remains insufficiently explained is why rejection risk varies so markedly among recipients who otherwise undergo similar surveillance and receive comparable calcineurin inhibitor-based immunosuppression.

The traditional explanatory framework for rejection has centered on donor–recipient immunobiology, perioperative injury, adherence, and therapeutic drug exposure [4,5,6]. Yet this model is incomplete. A growing body of transplant immunology shows that allograft rejection is not driven solely by adaptive immunity; innate immune activation, including dendritic-cell priming, NK-cell signaling, complement, and danger-signal amplification, shapes the threshold at which alloimmunity becomes clinically manifest. Importantly, these pathways are only incompletely suppressed by standard antirejection regimens. In that sense, transplantation is not simply a pharmacologic contest against T cells, but a dynamic equilibrium between alloantigen recognition and the surrounding inflammatory milieu [7,8].

Environmental exposures are a plausible but underexplored contributor to that inflammatory milieu [9,10]. In non-transplant populations, residential greenness is often associated with cardiovascular benefits, lower disease burden, and improved recovery after major cardiac interventions [11,12,13,14,15]. However, that literature usually treats “greenness” as a unitary beneficial exposure. In reality, biologically distinct landscape elements—tree canopy, low vegetation, stagnant water, traffic corridors, industrial land—are unlikely to exert equivalent immune effects. For a transplant recipient, the relevant issue may not be whether a neighborhood is broadly green, but whether it concentrates inhaled bioaerosols, moisture-associated microbial reservoirs, particulate irritants, or mixed land-use signatures that sustain low-grade immune activation.

This possibility is strengthened by emerging literature on transplant environments. Across solid-organ transplantation, post-transplant air pollution has been associated with poorer outcomes, including rejection-related risk signals, and recent work in lung transplantation suggests that environmental exposure early after transplantation may influence later graft dysfunction [16,17,18]. In kidney transplantation, a recent meta-analysis found that ambient particulate matter exposure was associated with a higher risk of rejection [19]. Still, the existing literature remains mainly focused on air-pollutant concentrations rather than on residential land-use architecture, and it is particularly sparse in heart transplantation.

To the best of our knowledge, the available literature [20], examined traffic-road proximity, not compositional neighborhood structure across concentric residential buffers.

Against that background, we designed a buffer-resolved compositional analysis of the residential environment after heart transplantation. Rather than testing dozens of land-use variables independently, we organized the exposure surface into five interpretable domains—trees, other green surroundings, roads, water, and industrial land—and examined how their relative burden changed from 300 m to 1000 m around the place of residence. This approach was intended to address two unresolved questions: first, whether rejection-associated environmental signals are scale-dependent; and second, whether apparently similar “green” exposures actually form biologically divergent patterns. Our working hypothesis was that rejection risk would be driven not by urbanicity per se, but by a multi-scale environmental architecture in which immediate tree-dominant surroundings, intermediate hydrologic features, and broader disturbed or industrial land each capture different forms of residual immune stress.

## 2. Methods

### 2.1. Study Design and Population

This was a retrospective single-center cohort study of adult heart transplant recipients undergoing routine post-transplant surveillance. Of 45 consecutive adult heart transplant recipients between January 2021 and January 2025 at a single tertiary center, 30 patients transplanted within the study period with complete follow-up data who did not change their residency during the observation period. At least one biopsy-linked geospatial data with laboratory findings were essential for the study. The final dataset comprised 30 recipients and 267 repeated observations.

Biopsies were performed according to a standard surveillance protocol at predefined intervals (e.g., 1 and 2 weeks, followed by 1, 2, 3, 4/5, 6, 9, and 12 months after heart transplantation), no additional biopsies triggered by clinical suspicion (e.g., decline in LVEF, arrhythmia, biomarker elevation) were performed in the analyzed population. The control biopsies that were performed after applying ACR therapy were not taken into the analysis.

### 2.2. Outcome Definition

The primary outcome was acute cellular rejection (ACR), defined as biopsy-confirmed rejection at each observation point. The analysis was performed at the visit level, allowing repeated measurements per patient. Only immunosuppression values measured prior to the biopsy were included in the analysis.

### 2.3. Environmental Exposure Assessment

Residential land-use data were extracted using geospatial buffers centered on each patient’s residence at 300 m, 500 m, 700 m, and 1000 m.

Residential addresses were geocoded, and land-use data were extracted as described in previous publications [21]. Circular buffers (300–1000 m) were constructed as previously described. No patient interviews were conducted.

Raw land-use classes were aggregated into five biologically interpretable domains:

- **Trees**: Forest, tree-covered areas.
- **Other green**: Shrubs, grassland, orchards, allotments.
- **Roads**: Road and transport surfaces.
- **Water**: Standing and flowing water.
- **Industrial**: Industrial, storage, extraction, and technical land.

Each domain was expressed as a percentage of the total buffer area.

In addition to clustered logistic regression models, a sensitivity analysis was performed using mixed-effects logistic regression with a random intercept for patient to explicitly account for within-subject correlation across repeated biopsy observations. Fixed effects included environmental domain burden (per 1 standard deviation increase) and log-transformed time since transplantation. Additional models were adjusted for age and sex. Full-model specifications and sensitivity analyses are presented in Supplementary Tables S1–S3.

### 2.4. Endomyocardial Biopsies

All endomyocardial biopsies (EMBs) were examined in the reference center as described in the previous report [22]. Biopsy fragments were fixed in a 4% buffered formaldehyde solution, then routinely dehydrated in an ethyl alcohol series and embedded in paraffin wax through xylene. Tissue slices, 5 microns thick, were stained with H&E (hematoxylin and eosin). The rejection grade was estimated using ISHLT’s working formulation [23]. Acute cellular rejection (ACR) was defined as stage 2 or higher.

### 2.5. Statistical Analysis

Associations were evaluated using logistic regression models with clustered standard errors at the patient level. Models were adjusted for log-transformed time since transplantation. Results are presented as odds ratios per 1 standard deviation increase in domain burden. Additional adjustments for age and sex were performed. The false discovery rate (FDR) correction for multiple comparisons has been performed.

This standardization allows comparison across domains and buffers despite differences in scale and variance.

Statistical analysis was performed using JASP statistical software (JASP Team, 2023, Version 0.18.1). *p* < 0.05 was considered statistically significant.

GenAI (https://generativeai.net/) was used to generate the graphical abstract.

## 3. Results

### 3.1. Cohort and Follow-Up Characteristics

The compositional analysis included 30 recipients with complete transplant date, biopsy follow-up, and geospatial land-use data. The median (IQR) number of observations per patient was 9 (8–10). These recipients contributed 267 longitudinal observations linked to scheduled surveillance biopsies. The observation window extended from day 31 to day 755 after transplantation, with a median follow-up time of 126 days. Acute cellular rejection occurred in 23 of 30 patients (76.7%), while 7 patients (23.3%) did not experience any rejection episode during follow-up. Across the analytic panel, 41 of 267 observations were ACR-positive. Rejection events were distributed across early (0–90 days) and intermediate (90–365 days) follow-up without a strictly monotonic decline. Group description was presented in Table 1.

### 3.2. Rejection Pattern Through the Observational Period

Visits corresponded to scheduled biopsy timepoints as per institutional protocol. Visit-level ACR rates were: Visit 1: 4/30, 13.3%; Visit 2: 7/30, 23.3%; Visit 3: 8/30, 26.7%; Visit 4: 3/30, 10.0%; Visit 5: 3/30, 10.0%; Visit 6: 3/29, 10.3%; Visit 7: 7/30, 23.3%; Visit 8: 7/30, 23.3%; Visit 9: 4/30, 13.3%; Visit 10: 2/29, 6.9%. Detailed information on follow-up time intervals, applied immunosuppressive therapies, immunosuppressive therapy serum levels, and echocardiographic left ventricular ejection fraction characteristics is presented in Table 2.

### 3.3. Clinical and Treatment-Related Variables

At baseline, no clinical variables were significantly associated with ACR at the patient level. Among time-varying variables, steroid dose was associated with ACR (OR 1.72, 95% CI 1.13–2.62; *p* = 0.011), whereas tacrolimus and mycophenolate levels were not significantly associated with rejection. Immunosuppressive measurements were restricted to values obtained prior to biopsy.

### 3.4. Environmental/OB-SE Land-Use Exposure Results

#### 3.4.1. Step 1. Buffer Composition

**Figure 1:**
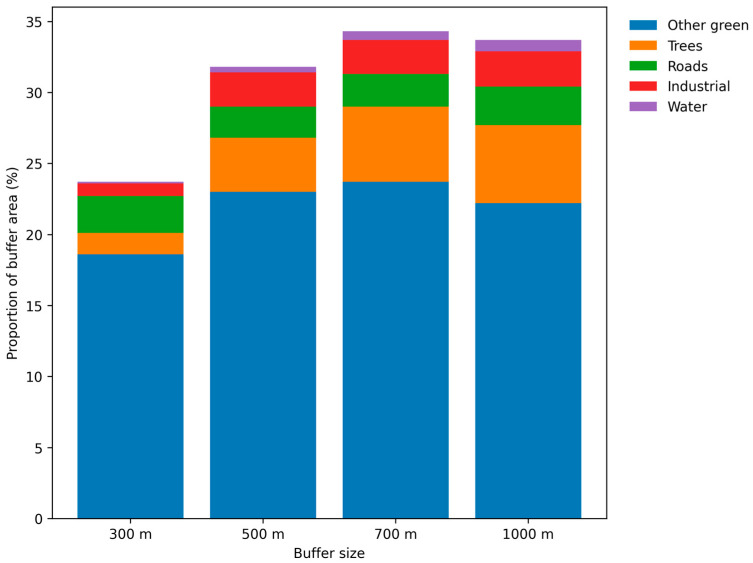
The proportions of analyzed buffers’ composition.

To make the buffer analysis interpretable, environmental variables were collapsed into five domains. Because the source geospatial registry stores land-use in areal units, the domain burden was converted to a percentage of the total buffer area. The descriptive pattern was highly structured: other green surroundings occupied the largest share of all buffers, whereas tree-dominant cover, water, roads, and industrial land each represented much smaller fractions. Crucially, the relative contributions of each domain varied with scale, with OR values representing standardized units (z-scores), not proportions, as shown in Table 3 and Figure 1.

#### 3.4.2. Step 2. Buffer Composition and Rejection Risk

**Figure 2:**
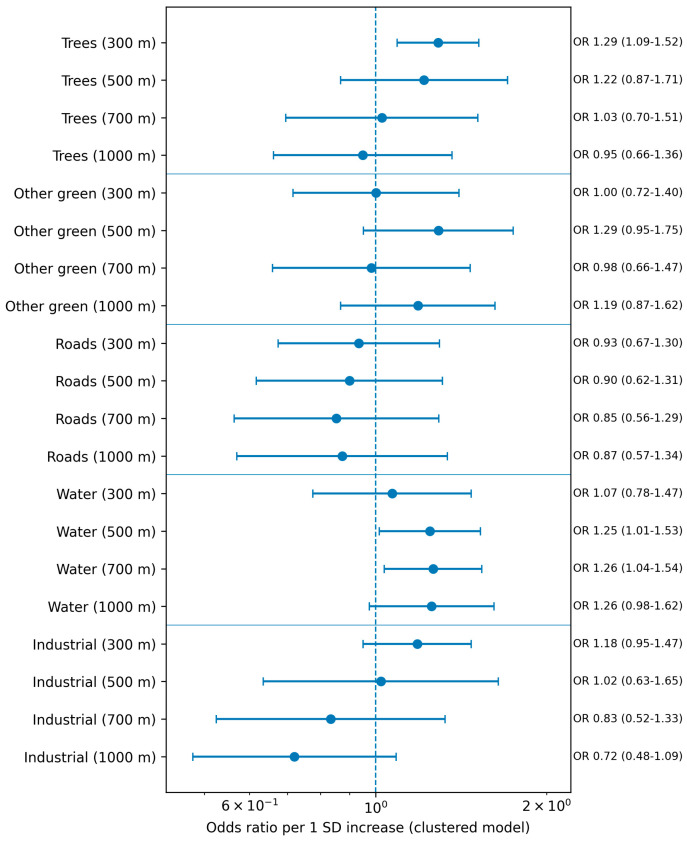
Forest plot of clustered logistic regression estimates across buffer sizes and environmental domains. Odds ratios are shown per 1 standard deviation increase in domain burden; horizontal lines indicate 95% confidence intervals.

Associations were modeled using repeated-measures logistic regression with clustering by recipient and adjustment for follow-up time. To allow comparison across domains and across scales, effects are presented as odds ratios per 1 standard deviation increase in the buffer-specific domain burden (Table 4, Figure 2).

Associations between environmental domains and ACR were strongest within the 300 m buffer. After false discovery rate (FDR) correction, only tree-dominant (OR 1.42, 95% CI 1.22–1.65, q = 0.010) and industrial land exposure (OR 1.50, 95% CI 1.28–1.76, q = 0.010) remained statistically significant.

At 500 m, tree exposure showed nominal significance (OR 1.39, *p* = 0.034) but did not survive FDR correction. No associations were observed beyond 700 m.

The potential impact of environmental factors on ACR risk, including the trees and industrial density in the analyzed buffers, was presented in Figure 2.

### 3.5. Sensitivity Analyses

To assess the robustness of the primary findings, additional analyses were performed using alternative model specifications.

In age- and sex-adjusted clustered logistic regression models, the association between tree-dominant exposure within the 300 m buffer and ACR remained statistically significant (OR ~ 1.3 per 1 SD increase, *p* < 0.01), while industrial exposure did not demonstrate a significant association.

In mixed-effects logistic regression models with a random intercept for patient, the magnitude of the tree-related effect was similar but did not reach statistical significance (OR ~ 1.3, *p* ≈ 0.06). Industrial exposure remained non-significant.

Across all sensitivity analyses, no environmental domain demonstrated consistent statistically significant associations beyond the 300 m buffer.

In clustered models adjusted for age and sex (Supplementary Table S2), the association between tree exposure and ACR remained significant (OR 1.32, *p* < 0.001), while industrial exposure was not significant.

In mixed-effects models accounting for within-patient correlation (Supplementary Table S3), the tree-related effect was attenuated (OR 1.29, *p* = 0.061), and no environmental domain demonstrated consistent statistical significance.

Across all supplementary analyses (Tables S1–S3), no associations were observed beyond the 300 m buffer.

## 4. Discussion

This study suggests a spatially constrained environmental signal associated with acute cellular rejection after heart transplantation. The central finding is that rejection risk was linked to the immediate residential environment within a 300 m radius, whereas no consistent associations were observed at larger spatial scales. This pattern suggests that environmentally mediated influences on rejection are not driven by general neighborhood characteristics, but rather by highly localized exposure processes.

The defining feature of the observed associations is their spatial restriction. Signals were present at 300 m, weakened at 500 m, and absent beyond 700 m across all models. This gradient argues against a broad ecological or socioeconomic explanation and instead supports a model of direct, repeated exposure within the immediate residential microenvironment. Importantly, the absence of associations at larger spatial scales should be interpreted as a key negative finding, indicating that any environmental contribution to rejection risk is likely to operate at short-range exposure distances rather than through generalized environmental context.

While several signals were observed at larger spatial scales in unadjusted analyses, none remained significant after FDR correction. This finding underscores the importance of accounting for multiple testing in multi-domain, multi-scale environmental analyses and suggests that proximal environmental signals are less likely to represent chance associations than those observed at larger spatial scales. The consistency of findings across supplementary analyses was limited, with attenuation observed in the mixed-effects models (Supplementary Table S3), suggesting caution in interpretation.

Within this localized framework, two domain-specific signals were observed. Tree-dominant environments and exposure to industrial land were associated with increased odds of rejection in clustered models, although only the tree-related signal persisted across sensitivity analyses. In mixed-effects models accounting for within-subject correlation, the magnitude of the tree association remained similar but did not reach statistical significance, while the industrial signal was not consistently observed. These findings indicate that the detected associations are sensitive to model specification and should be interpreted as hypothesis-generating rather than definitive.

A key conceptual implication of these results is the divergence between vegetation subtypes. While residential greenness is generally associated with favorable cardiovascular outcomes in non-transplant populations, the present analysis suggests that tree-dominant environments may represent a biologically distinct exposure in immunosuppressed patients. Other green surroundings—including shrubs, grassland, and managed vegetation—did not demonstrate similar associations. This distinction challenges the assumption that greenness can be treated as a homogeneous protective exposure and suggests that vegetation phenotype, rather than overall green coverage, determines biological relevance in the transplant setting. The attenuation of associations with increasing buffer size argues against a purely ecological or socioeconomic explanation and instead supports a model of direct, repeated environmental exposure. Our previous publications identified the role of buffer composition in the progression of coronary artery disease [21].

A plausible mechanistic framework linking local environmental composition to rejection involves innate immune activation at mucosal surfaces. Tree-dominant environments are associated with increased concentrations of airborne bioaerosols, including pollen and fungal spores, which are known to activate dendritic cells and amplify cytokine signaling. In transplant recipients, these upstream inflammatory pathways are not fully suppressed by standard immunosuppressive regimens, potentially lowering the threshold for alloimmune activation. In parallel, industrial land may contribute particulate and inorganic exposures that promote systemic inflammation. Together, these exposures may act as non-specific immune amplifiers, modulating rejection risk without directly triggering alloimmunity. Tree-rich environments are associated with higher concentrations of airborne pollen, fungal spores, and organic particulate matter [24,25,26]. These exposures are well-established triggers of innate immune activation at mucosal surfaces, promoting dendritic cell maturation, cytokine release, and amplification of inflammatory signaling. In the context of transplantation, such upstream activation is particularly relevant because it operates outside the primary targets of calcineurin inhibition and antiproliferative therapy.

The spatial attenuation of associations with increasing buffer size further supports this interpretation. If the observed effects were driven by broader environmental or socioeconomic factors, one would expect signal persistence or strengthening at larger spatial scales. Instead, the rapid loss of association beyond 300–500 m suggests that exposure intensity and proximity—rather than cumulative environmental burden—are the dominant determinants.

Equally important are the negative findings. Roads and other green surroundings did not demonstrate consistent associations with rejection, and no environmental domain showed robust effects beyond the immediate residential buffer. These results argue against a simplified interpretation based on urbanization gradients or general environmental quality and instead support a more specific, domain-dependent framework of environmental influence. Industrial and disturbed land surfaces act as sources of particulate matter, inorganic dust, and mixed environmental pollutants [27,28]. Unlike large-scale air pollution metrics, which reflect regional exposure, the present signal appears confined to micro-environmental proximity, suggesting that local exposure intensity may be more relevant than ambient averages.

Conceptually, these findings extend the current understanding of rejection risk after heart transplantation. While traditional models emphasize immunological compatibility and pharmacological suppression, the present data suggest that a localized environmental context may act as an additional modifier of immune activation. This perspective introduces a potential external dimension to transplant risk that is not captured by standard clinical variables.

The clinical implications of these findings remain uncertain. The observed associations do not support immediate changes in patient management, but they provide a testable framework for future studies integrating environmental exposure data with transplant immunology. Larger, multicenter cohorts and studies incorporating direct measurements of airborne exposures will be required to determine whether these signals reflect causal relationships and can be translated into risk stratification or preventive strategies.

### Limitations

This study should be interpreted in the context of several limitations. The small number of patients limits generalizability and may introduce instability in estimates. While the longitudinal structure and repeated-measures design increase analytical robustness, the possibility of type I and type II errors cannot be ruled out, particularly given the number of environmental domains and buffer levels examined. Environmental exposure was assessed using land-use composition rather than direct measurements of biological or chemical exposure. The study, therefore, cannot distinguish which specific components of these environments are causally responsible for the observed associations.

The exposures were assumed to be stable over time and to vary by residential location, without accounting for mobility or temporal variation in environmental conditions. The residential address was assumed to remain stable during follow-up, potentially leading to exposure misclassification.

Finally, multiple comparisons were performed across several environmental domains and spatial scales. Although the observed patterns were internally consistent with known mechanisms of innate immune activation, the potential for chance findings cannot be fully excluded, and external validation in independent cohorts is required. Clustered logistic regression was used, mixed-effects models with random intercepts may provide a more robust framework and should be considered in future studies.

## 5. Conclusions

Residential environmental composition may be associated with acute cellular rejection after heart transplantation in a scale-dependent manner, with signals confined to the immediate residential environment. Tree-dominant exposure within 300 m showed an association in clustered models; however, this finding was attenuated in mixed-effects sensitivity analyses. No environmental domain demonstrated consistent associations across all analytical approaches.

These results should be considered exploratory and hypothesis-generating, warranting validation in larger, multi-center cohorts with more robust statistical modeling.

## Figures and Tables

**Table 1:** Group characteristics.

Variables	Value
Sex (males/females)	25/5
Age (years) (median) (Q1–Q3))	48.5 (38.3–60.0)
Body mass index (BMI) (kg/m^2^) (median) (Q1–Q3))	25.3 (23.5–28.7)
Primary diagnosis:	
Dilated cardiomyopathy (*n* (%))	17 (57)
Ischemic cardiomyopathy (*n* (%))	13 (43)
Co-morbidities:	
Arterial hypertension (*n* (%))	10 (33)
Diabetes mellitus (*n* (%))	5 (17)
Chronic obstructive pulmonary disease (COPD) (*n* (%))	3 (10)
Peripheral artery disease (PAD) (*n* (%))	0 (0)
Stroke (*n* (%))	2 (7)
Kidney disease (GFR below 60 mL/min/m2) (*n* (%))	6 (20)
Dyslipidemia (*n* (%))	9 (30)
	
Lifestyle factors:	
Cigarette use (*n* (%))	6 (20)
Alcohol use (*n* (%))	1 (3)
Operative characteristics:	
Lower–Shumway technique (*n* (%))	30 (100)
Cold Ischemic time (minutes) (median) (Q1–Q3)	225 (190–252)
Median follow-up time (days) (median) (Q1–Q3)	126 (62.5–203)

Abbreviations: kg—kilogram, m^2^—square meter, n—number, %—percentage, Q—quartile.

**Table 2:** Immunosuppression and EMB results in the analyzed time intervals.

Variables	Time-1	Time-2	Time-3	Time-4	Time-5	Time-6	Time-7	Time-8	Time-9	Time-10
Days after HTX (median (Q1–Q3))	8 (6–10)	19 (16–28)	35 (30–42)	61 (49–63)	91 (81–98)	123 (105–128)	154 (136–163)	182 (158–193)	273 (192–280)	365 (276–371)
Therapy dosing:										
Tacrolimus (mg/day)	8 (6–10)	8 (5–10)	6 (4–8)	6 (5–8)	5 (4–7)	5.0 (3.0–6.0)	5 (3–6)	4.5 (3.0–6.0)	3.0 (2.0–5.0)	4.0 (2.0–5.5)
MPA (g/day)	2 (2.0–3.0)	2.5 (2.0–3.0)	2.5 (1.625–3.0)	2 (1.25–2.5)	1.5 (1.5–2.0)	1.5 (1.3–2.0)	1.5 (1.0–2.0)	1.5 (1.0–2.0)	1.3 (1.0–1.6)	1.0 (1.0–1.5)
Steroids (mg/day)	55 (30–65)	55 (30–65)	45 (30–65)	35 (15–50)	25 (15–40)	17.5 (10–35)	20 (10–25)	10 (5–30)	5 (2.5–20)	5 (2.5–10)
Immunosuppression levels:										
Tacrolimus (C-0) (ng/mL) (median (Q1–Q3))	12 (9–15)	14 (13–16)	14 (12–18)	15 (11–18)	13 (11–16)	13 (10–14)	17 (11–17)	12 (11–14)	14 (10–17)	11 (10–15)
MPA (C-0) (ug/mL) (median (Q1–Q3))	2.3 (1.5–3.5)	2.2 (1.5–3.6)	2.9 (1.8–4.8)	2.8 (1.9–3.9)	2.8 (1.7–3.8)	2.3 (1.7–3.3)	2.2 (1.2–3.3)	2.1 (1.7–2.4)	2.1 (1.5–2.6)	1.8 (1.5–2.2)
MPA (C-30) (ug/mL) (median (Q1–Q3))	3.0 (3.4–9.6)	3.9 (2.2–7.7)	5.3 (4.2–8.4)	4.7 (3.4–7.6)	5.4 (3.9–8.1)	6.9 (3.9–9.0)	5.5 (4.3–8.3)	4.9 (3.3–8.0)	4.8 (3.3–8.0	6.2 (4.5–8.8)
MPA (C-120) (ug/mL) (median (Q1–Q3))	4.5 (3.1–6.3)	5.5 (2.7–9.4)	5.3 (4.8–7.8)	5.7 (4.2–7.7)	5.7 (3.0–10.0))	6.5 (5.7–8.0)	5.0 (3.8–6.7)	4.5 (3.0–6.0)	3.9 (3.2–5.3))	4.1 (3.1–5.5)
Echocardiography:										
LVED (mm) (median (Q1–Q3))	46 (42–48)	45 (43–47)	45 (43–48)	43 (41–46)	45 (42–49)	45 (42–47)	45 (42–48)	43 (42–48)	45 (43–49)	46 (42–48)
IVs (mm) (median (Q1–Q3))	12 (10–13)	12 (11–14)	11 (10–12)	12 (11–13)	10 (10–13)	12 (11–13)	11 (11–12)	11 (10–12)	11 (10–12)	10 (10–12)
LVEF (%) (median (Q1–Q3))	60 (60–65)	60 (60–65))	65 (65–68)	68 (64–68)	65 (65–68)	65 (65–68)	65 (65–68)	65 (65–68)	65 (65–68)	65 (64–68)
Biopsy-proven rejection (n (%))	4 (13.3)	7 (23.3)	8 (26.7)	3 (10.0)	3 (10.0)	3 (10.0)	7 (23.3)	7 (23.3)	4 (13.3)	2 (6.9)

Abbreviations: C-0—serum concentration before morning dose; C-30—serum concentration 30 min after morning dose; C-120—serum concentration 120 min after morning dose; IVs—interventricular septum; g—grams; HTX—heart transplantation; LVED—left ventricular ejection fraction; LVEF—left ventricular ejection fraction; mg—milligrams; MPA—mycophenolate mofetil acid; n—number; Q—quartiles.

**Table 3:** Residential buffers composition in the analyzed population.

Domain	300 m	500 m	700 m	1000 m
Trees	0.015 (0.000–0.064)	0.038 (0.006–0.072)	0.053 (0.009–0.111)	0.055 (0.030–0.175)
Other green	0.186 (0.134–0.296)	0.230 (0.116–0.288)	0.237 (0.139–0.301)	0.222 (0.131–0.315)
Roads	0.026 (0.000–0.051)	0.022 (0.010–0.057)	0.023 (0.010–0.057)	0.027 (0.009–0.051)
Water	0.001 (0.000–0.008)	0.004 (0.000–0.015)	0.006 (0.001–0.050)	0.008 (0.001–0.042)
Industrial	0.009 (0.000–0.031)	0.024 (0.001–0.058)	0.024 (0.005–0.063)	0.025 (0.005–0.061)

Abbreviations: m—meter.

**Table 4:** Association between buffer composition and ACR risk. Odds ratio per 1 SD increase in domain burden.

Domain	300 m OR (95% CI)	*p*	FDR q	500 m OR (95% CI)	*p*	FDR q	700 m OR (95% CI)	*p*	FDR q	1000 m OR (95% CI)	*p*	FDR q
Trees	**1.42 (1.22–1.65)**	**<0.001**	**0.010**	**1.39 (1.03–1.88)**	**0.034**	0.227	1.17 (0.82–1.67)	0.378	0.612	0.96 (0.68–1.37)	0.838	0.838
Other green	1.03 (0.74–1.43)	0.843	0.887	1.26 (0.96–1.67)	0.100	0.286	0.93 (0.63–1.37)	0.714	0.840	1.14 (0.84–1.55)	0.398	0.612
Roads	0.88 (0.63–1.23)	0.464	0.628	0.75 (0.51–1.11)	0.148	0.300	0.70 (0.46–1.05)	0.088	0.286	0.74 (0.48–1.12)	0.150	0.300
Water	0.98 (0.59–1.65)	0.946	0.946	1.28 (0.99–1.64)	0.057	0.285	1.22 (0.97–1.52)	0.091	0.286	1.16 (0.89–1.51)	0.262	0.476
Industrial	**1.50 (1.28–1.76)**	**<0.001**	0.010	1.18 (0.75–1.85)	0.471	0.628	0.91 (0.56–1.48)	0.702	0.840	0.74 (0.50–1.10)	0.140	0.300

Abbreviations: CI—confidence interval, m—meter, OR—odds ratio.

## Data Availability

Data supporting the reported results are available upon reasonable request from the corresponding author via e-mail.

## Supplementary Materials

The following supporting information can be downloaded at: https://www.mdpi.com/article/10.3390/jcm15093272/s1, Table S1: Association between environmental domains and ACR risk using clustered logistic regression models; Table S2: Association between environmental domains and ACR risk using clustered logistic regression models adjusted for age, sex, and time since transplantation; Table S3: Association between environmental domains and ACR risk using mixed-effects logistic regression with random patient intercepts.
