# Supplementary material for: Residential Environmental Composition and Risk of Acute Cellular Rejection After Heart Transplantation: A Multi-Scale Buffer Analysis
Source: J Clin Med. 2026 Apr 24;15(9):3272. doi: 10.3390/jcm15093272 (PMC13163871; doi:10.3390/jcm15093272)
Supplement: Supplementary file 1 [file jcm-15-03272-s001.zip › jcm-4253989-supplementary.pdf]

Supplementary Table S1.

Association between environmental domains and ACR risk using clustered logistic regression models (adjusted for log-transformed time since transplantation)

Domain	Buffer	OR (per 1 SD)	95% CI	p-value	FDR q
Trees	300 m	1.29	1.09 – 1.52	0.0027	0.013
	500 m	1.18	0.96 – 1.45	0.11	0.28
	700 m	1.10	0.88 – 1.38	0.40	0.61
	1000 m	0.97	0.78 – 1.21	0.81	0.84
Other green	300 m	1.04	0.80 – 1.36	0.77	0.88
	500 m	1.19	0.95 – 1.50	0.13	0.29
	700 m	0.95	0.72 – 1.26	0.74	0.84
	1000 m	1.08	0.84 – 1.39	0.55	0.61
Roads	300 m	0.90	0.68 – 1.20	0.48	0.63
	500 m	0.82	0.61 – 1.10	0.19	0.30
	700 m	0.76	0.56 – 1.04	0.09	0.29
	1000 m	0.80	0.58 – 1.12	0.20	0.30
Water	300 m	1.02	0.65 – 1.60	0.94	0.95
	500 m	1.22	0.98 – 1.53	0.07	0.29
	700 m	1.18	0.96 – 1.45	0.12	0.29
	1000 m	1.12	0.90 – 1.39	0.31	0.48
Industrial	300 m	1.18	0.95 – 1.47	0.13	0.30
	500 m	1.10	0.85 – 1.42	0.47	0.63
	700 m	0.94	0.70 – 1.25	0.67	0.84
	1000 m	0.88	0.67 – 1.16	0.36	0.48

Abbreviations: OR – odds ratio; CI – confidence interval; SD – standard deviation.

All models are expressed per 1 standard deviation increase in environmental domain burden. Clustered models account for repeated observations using clustered standard errors at the patient level. Mixed-effects models include a random intercept for patient.

Supplementary Table S2

Association between environmental domains and ACR risk using clustered logistic regression models adjusted for age, sex, and time since transplantation

Domain	Buffer	OR (per 1 SD)	95% CI	p-value
Trees	300 m	1.32	1.14 – 1.53	<0.001
	500 m	1.21	1.00 – 1.47	0.051
	700 m	1.11	0.89 – 1.39	0.34
	1000 m	0.98	0.79 – 1.22	0.86
Other green	300 m	1.06	0.81 – 1.39	0.68
	500 m	1.22	0.96 – 1.55	0.10
	700 m	0.96	0.73 – 1.27	0.79
	1000 m	1.10	0.85 – 1.43	0.47
Roads	300 m	0.91	0.69 – 1.21	0.53
	500 m	0.83	0.62 – 1.12	0.23
	700 m	0.77	0.57 – 1.05	0.10
	1000 m	0.82	0.59 – 1.14	0.24
Water	300 m	1.03	0.66 – 1.62	0.90
	500 m	1.24	0.99 – 1.56	0.06
	700 m	1.19	0.97 – 1.47	0.10
	1000 m	1.14	0.92 – 1.41	0.23
Industrial	300 m	1.12	0.91 – 1.39	0.28
	500 m	1.08	0.83 – 1.41	0.55
	700 m	0.93	0.70 – 1.24	0.62
	1000 m	0.87	0.66 – 1.15	0.33

Abbreviations: OR – odds ratio; CI – confidence interval; SD – standard deviation.

All models are expressed per 1 standard deviation increase in environmental domain burden. Clustered models account for repeated observations using clustered standard errors at the patient level. Mixed-effects models include a random intercept for the patient.

Supplementary Table S3

Association between environmental domains and ACR risk using mixed-effects logistic regression with random patient intercepts

**Domain**	**Buffer**	**OR (per 1 SD)**	**95% CI (approx.)**	**p-value**
Trees	300 m	1.29	0.99 – 1.68	0.061
	500 m	1.16	0.91 – 1.49	0.22
	700 m	1.08	0.82 – 1.43	0.58
	1000 m	0.96	0.73 – 1.27	0.78
Other green	300 m	1.05	0.78 – 1.41	0.74
	500 m	1.18	0.90 – 1.55	0.23
	700 m	0.94	0.68 – 1.29	0.69
	1000 m	1.07	0.80 – 1.43	0.65
Roads	300 m	0.92	0.67 – 1.25	0.60
	500 m	0.84	0.60 – 1.17	0.30
	700 m	0.79	0.55 – 1.13	0.20
	1000 m	0.83	0.58 – 1.19	0.31
Water	300 m	1.01	0.59 – 1.71	0.97
	500 m	1.20	0.92 – 1.56	0.17
	700 m	1.16	0.91 – 1.49	0.23
	1000 m	1.10	0.85 – 1.43	0.46
Industrial	300 m	1.17	0.89 – 1.53	0.26
	500 m	1.08	0.79 – 1.47	0.62
	700 m	0.92	0.65 – 1.30	0.64
	1000 m	0.86	0.62 – 1.20	0.38

Abbreviations: OR – odds ratio; CI – confidence interval; SD – standard deviation.

All models are expressed per 1 standard deviation increase in environmental domain burden. Clustered models account for repeated observations using clustered standard errors at the patient level. Mixed-effects models include a random intercept for the patient.
